# Sequencing Choices and Real-World Clinical Management in Advanced Grade 2/3 GEP-NET Treatment: The Emerging Role of PRRT

**DOI:** 10.3390/cancers17183008

**Published:** 2025-09-15

**Authors:** Aman Chauhan, Thorvardur R. Halfdanarson, Namrata Vijayvergia

**Affiliations:** 1 Sylvester Comprehensive Cancer Center, University of Miami Health System, Miami, FL 33136, USA; 2 Mayo Clinic, Rochester, MN 55905, USA; 3 Fox Chase Cancer Center, Philadelphia, PA 19111, USA; namrata.vijayvergia@fccc.edu

**Keywords:** advanced GEP-NETs, PRRT, treatment sequencing

## Abstract

The prognosis of high-grade gastroenteropancreatic neuroendocrine tumors (GEP-NETs) is highly variable and reported median overall survival remains low. There is no established standard of care for patients with higher grade 2 (G2) (Ki-67 ≥ 10% and ≤ 20%) and grade 3 (G3) (Ki-67 ≥ 20% and ≤ 55%) GEP-NETs and most of the current treatment regimens, particularly for G3 NETs, are extrapolated from lower-grade GEP-NET treatments or based on data from small retrospective studies. Robust evidence to support treatment recommendations for high-grade NETs, especially in the first-line (1L) setting, is limited and the optimal treatment sequence has not been clearly defined. This review summarizes the latest literature on sequencing of therapies, identifies patient selection considerations for utilizing peptide receptor radionuclide therapy (PRRT), and offers data-driven expert opinions and clinical practice recommendations in high-grade GEP-NETs. As authors, we recommend that all patients with well-differentiated, higher G2 and G3 NETs are evaluated with [^68^Ga]Ga-DOTA-peptide positron emission tomography (PET) for baseline somatostatin receptor expression and if positive, a 1L treatment option of PRRT should be considered, especially in clinically stable patients. Somatostatin analog therapy may benefit patients with low-volume, indolent disease, and chemotherapy is usually a better fit for patients in visceral crisis, in need of urgent treatment, or with no access to [^68^Ga]Ga-DOTA-peptide PET. In complex cases, a robust discussion at a multidisciplinary NET tumor board can be beneficial.

## Simple Summary

For higher-grade, advanced gastroenteropancreatic neuroendocrine tumors, there is not a clear standard treatment sequence, and many current approaches are based on lower-grade cases or small retrospective studies. The importance of using specific imaging tests to guide treatment decisions for these tumors is highlighted and the potential of peptide receptor radionuclide therapy, a targeted therapy, as a first-line option for certain patients is discussed. Other treatments like somatostatin analogs may help some patients with slower-growing tumors, while chemotherapy is often needed for those in need of urgent treatment. Overall, this review aims to provide updated recommendations for the sequence of treatments for these complex tumors.

## 1. GEP-NETs Overview

### 1.1. GEP-NET Background Information and GEP-NET World Health Organization Classification

Neuroendocrine neoplasms (NENs) arise from the neuroendocrine system and are classified by the status of their differentiation, with the two major categories of clinical and prognostic significance represented by neuroendocrine tumors (NETs; well differentiated) and neuroendocrine carcinomas (NEC; poorly differentiated) [1,2]. NETs primarily occur in the lungs, digestive tract, and pancreas with the latter two groups collectively termed gastroenteropancreatic NETs (GEP-NETs) [3]. NETs of gastrointestinal origin represent the second-most prevalent neoplasm of gastrointestinal origin after colorectal cancer [1]. Pancreatic NETs (pNETs) are uncommon, accounting for only about 2% of all pancreatic malignancies but the incidence has increased threefold in the last 2 decades, mostly because of incidental diagnoses at earlier stages [1,4,5].

Although regularly classified as a rare malignancy, the incidence and prevalence of GEP-NETs have been on the rise over the past few decades, likely driven by increases in clinician awareness, improved capabilities of diagnostic endoscopies, as well as the long survival of patients with localized and/or low-grade disease [1,6,7,8,9,10,11]. GEP-NETs are graded low grade (G1), intermediate grade (G2), or high grade (G3), while thoracic well-differentiated NETs are classified as typical or atypical carcinoids based on their mitotic rate [12]. A distinct G3 NET category was defined in 2019 by the World Health Organization owing to its different biology and prognosis as compared with the poorly differentiated NECs. G3 NET refers to morphologically well-differentiated tumors with a mitotic count > 20 per 2 mm^2^ and/or a Ki-67 proliferation index > 20% [1,13]. While the same mitotic count and Ki-67 criteria for G3 NETs apply to NECs, by definition, NECs have poorly differentiated morphology and are frequently associated with extensive tumor necrosis and a Ki-67 index > 55% [1]. Discussion about NECs is outside the scope of this review and has been discussed elsewhere in the literature [14,15]. As tumors of endocrine cell origin, NETs are also classified as functioning (hormonal hypersecretors) or non-functioning, with functioning tumors commonly associated with a range of characteristic hormonal syndromes [12].

**Figure 1:**
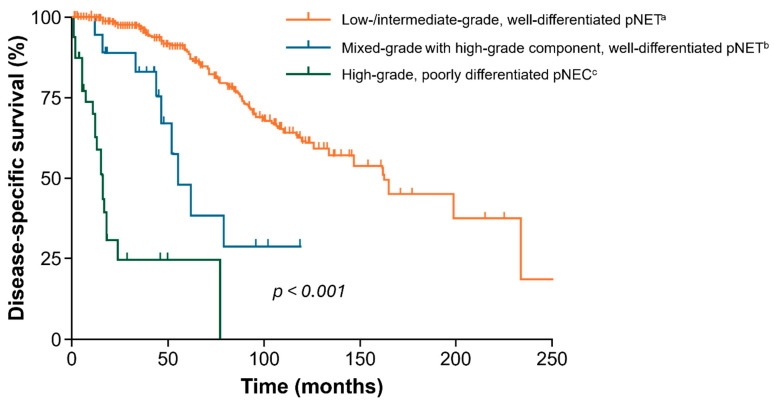
Disease-specific survival of stage-matched, well-differentiated pNETs with or without high-grade component and poorly differentiated pNECs. Figure adapted with permission from Tang et al. [22] ^a^ *n* = 329 patients; ^b^ *n* = 21 patients; ^c^ *n* = 35 patients. pNEC = pancreatic neuroendocrine carcinoma; pNET = pancreatic neuroendocrine tumor.

Even though G3 NETs and NECs are often readily distinguished by morphology, the diagnosis may be challenging even for NET specialists, particularly with small biopsy samples [16,17,18,19]. The prognosis of NENs is associated with their histologic grade, differentiation, and stage of disease, and for NETs specifically, the median overall survival (OS) can range from > 30 years for localized NETs to 12 months for distant NETs [2]. In recent years, however, more patients are being diagnosed during the earlier stages of their disease, and this has correlated with significant improvements in survival [4,20]. In a retrospective analysis of patients with high-grade (Ki-67 > 20%) NETs, a significantly better OS was observed in patients with Ki-67 ≤ 30% [21]. Well-differentiated NETs with a high-grade component are associated with lower survival rates than G1/G2, but they still show significantly better prognosis than poorly differentiated NECs (Figure 1) [22]. In general, the prognosis for metastatic G3 NET is highly variable and the reported median OS ranges from 3 to 8 years [23]. However, data are generally scarce for G3 GEP-NETs and it has been noted that incidence rates of G3 GEP-NETs are probably underestimated in the literature [24]. Overall, GEP-NETs are highly heterogeneous malignancies that remain orphan diseases, with both their diagnosis and optimal treatment having been long neglected [25]. Recent clinical advances show significant improvements in the diagnosis and treatment of GEP-NETs, but clinical outcomes and survival results remain unsatisfactory, and further innovation is needed in the diagnosis, prognosis, and treatment of these pathologies [25].

### 1.2. High-Grade GEP-NET Current Treatment Landscape Overview

Given the relative infrequency of high-grade NENs, there are few prospectively collected data to guide practitioners as to optimal patient management, and best practices are therefore based largely upon expert opinion [17]. Compounding this problem, given the relatively recent recognition of G3 NETs as a subset of G3 NENs, many older studies do not adequately distinguish G3 NETs from NECs, and the interpretation of the results is further limited due to G3 NETs being only a small proportion of the total study populations [17].

For patients with localized G3 NET, the goals of surgery are oncologic resection of the primary tumor and regional lymph nodes [17]. Chemotherapy is extensively used for G3 NETs, with temozolomide, fluoropyrimidines, etoposide, and platinum being the most common [13]. There are no high-quality data regarding the use of adjuvant chemotherapy for patients with resected NETs of any grade or stage, despite the risk of recurrence, especially of G3 NETs. In advanced/unresectable or recurrent GEP-NETs, pharmacologic somatostatin analog (SSA) treatment is common in clinical practice [26]. But very few data exist to guide the use of SSAs in somatostatin receptor (SSTR)-positive G3 NETs from the standpoints of either symptom control or cancer growth [17]. Targeted therapy in G3 NET is not well established, although everolimus has been considered in patients with progressive and refractory high-grade NETs of both pancreatic and bowel origin, as well as sunitinib for G3 pNET, particularly in patients with favorable biology [17]. Emerging data from the CABINET trial support the use of cabozantinib as a potential treatment option for patients with advanced extrapancreatic NETs (epNETs) or pNETs who have progressed on prior therapies; however, results should be interpreted with caution for higher-grade NETs, as only 6% and 13% of patients with G3 epNET and pNET, respectively, were randomized to cabozantinib [27]. Peptide receptor radionuclide therapy (PRRT) is a molecularly targeted radiation therapy involving the administration of a radiolabeled peptide, which targets specific receptors (e.g., SSTRs) [16]. Approximately 90% of GEP-NETs express SSTR2 [28] and present an opportunity to be candidates for SSTR-targeted therapy, including [^177^Lu]Lu-DOTATATE. Based on the NETTER-1 (NCT01578239) trial of patients with G1/G2 midgut NETs, [^177^Lu]Lu-DOTATATE PRRT has been approved in the U.S. and Europe for the treatment of SSTR-positive GEP-NETs [29,30]. NETTER-2 (NCT03972488) was the first phase 3 study that reported results for PRRT first-line (1L) therapy in any cancer population and the first randomized study of any therapy for patients with well-differentiated high-grade GEP-NETs. In NETTER-2, of patients randomized to [^177^Lu]Lu-DOTATATE, 54.3% had pNET and 45.7% had other GEP-NETs, while 65.6% of tumors were G2 and 34.4% were G3 [31].

### 1.3. Outcomes from Current Treatments for Patients with G2/G3 GEP-NETs

Surgical resection for locoregional control, and possible cure, is recommended for appropriate patients, yielding a median survival of 43–55 months in patients with G3 NET [17]. Carefully selected patients with metastatic G3 NETs may benefit from resection of liver metastases [32]. There is scarce evidence on the use of SSAs in these patients and most data on treatment outcomes of any therapy in G3 NETs come from retrospective studies [33,34,35,36,37]. More data are clearly needed to help guide clinicians in the management of these newly defined tumors [38].

An overview of key safety and efficacy data of current GEP-NET–targeted treatments is included in Table 1. Results from the PROMID study of patients with G1/G2 midgut NETs treated with 1L octreotide long-acting release (LAR) reported a median progression-free survival (PFS) of 14.3 months [39]. In the CLARINET study, extended-release lanreotide (aqueous gel formulation) was associated with significantly improved median PFS (not reached) compared with placebo (18.0 months; *p* < 0.001) among patients with metastatic, G1/G2, SSTR-positive GEP-NET (Ki-67 < 10%) [40]. A retrospective study of 1L SSA therapy in pNETs with Ki-67 ≥ 10% (68 G2 and five G3) revealed a median PFS of 4 months for G3 patients, suggesting a limited role of SSA therapy in G3 pNET [41]. As reported in the RADIANT-3 [42] and NCT00428597 [43] trials, second-line (2L) treatment of G1/G2 pNETs with everolimus or sunitinib resulted in median PFS of 11.0 and 11.4 months, respectively. Patients with G3 GEP-NENs undergo chemotherapy for tumor growth control, symptom alleviation, and OS improvement [25]. However, despite the variety of available chemotherapeutic strategies, there is no standard approach [25]. A recent study of patients with G1/G2 pNETs showed that 2L capecitabine and temozolomide (CAPTEM) was associated with improved PFS compared with temozolomide monotherapy (22.7 months vs. 14.4 months) [44]. In a study of 30 patients with G3 NEN (20% < Ki-67 < 55%) receiving CAPTEM (NCT03079440), the best overall response was complete response (CR) in one (3.3%), partial response (PR) in eight (26.7%), stable disease (SD) in 14 (46.7%), and progressive disease (PD) in four (13.3%) patients [45]. In NETTER-2, out of 151 patients with high-grade GEP-NETs (10% ≤ Ki-67 ≤ 55%) who received 1L [^177^Lu]Lu-DOTATATE, CR was seen in eight (5%), PR in 57 (38%), SD in 72 (48%), and PD in eight (5%) patients, while median PFS was 22.8 months [31]. It has been reported that responses to temozolomide-based therapy in G3 NEN patients are more common in the 1L setting, and in patients with pancreatic NENs [34].

**Table 1:** Efficacy and safety data of common GEP-NET treatments.

Trial	Type	Grade	Therapy	Line	mPFS (Months)	ORR (%, 95% CI)	Safety Events
SSAs							
CLARINET [40]	GEP-NET	G1/G2	Lanreotide	1L/2L	NR	NR	Hyperglycemia, cholelithiasis
PROMID [39]	Midgut NET	G1/G2	Octreotide LAR	1L	14.3	NR	GI events, hematologic events, fatigue, fever, bile stones
PRRT							
NETTER-1 [46]	Midgut NET	G1/G2	[^177^Lu]Lu-DOTATATE	2L	NR	18 (10–25)	Hematologic toxicity, nausea, vomiting
NETTER-2 [31]	GEP-NET	G2/G3	[^177^Lu]Lu-DOTATATE	1L	22.8	43 (35–51)	Hematologic toxicity, nausea, diarrhea, abdominal pain
Rotterdam [47]	GEP-NET	G1–G3	[^177^Lu]Lu-DOTATATE	2L	40	46 (NR)	Hematologic toxicities, nausea, vomiting, abdominal cramps
COMPETE [48]	GEP-NET	G1/G2	[^177^Lu]Lu-edotreotide	1L/2L	23.9	NR	NR
OCLURANDOM [49]	pNET	Advanced progressive	[^177^Lu]Lu-DOTATATE	≥ 2L	20.7	63	Hematologic toxicity, GI events, fatigue, hypertension, CKD, second cancers
mTOR inhibitors							
RADIANT-3 [42]	pNET	G1/G2	Everolimus	2L	11	5 (confirmed responses)	Stomatitis, rash, diarrhea, fatigue
RADIANT-4 [50]	GEP-NET	G1–G3	Everolimus	2L	11	2 (confirmed responses)	Stomatitis, diarrhea, infections, anemia, fatigue
TKIs							
NCT00428597 [43]	pNET	G1/G2	Sunitinib	2L	11.4	9.3	Diarrhea, nausea, vomiting, asthenia
CABINET [27]	NETs	G1–G3	Cabozantinib	3L	8.4 (extrapancreatic) 13.8 (pancreatic)	5 (extrapancreatic) 19 (pancreatic)	Hypertension, fatigue, diarrhea, thromboembolic events

1L = first line; 2L = second line; 3L = third line; CI = confidence interval; G = grade; CKD, chronic kidney disease; GEP-NET = gastroenteropancreatic neuroendocrine tumor; GI = gastrointestinal; LAR = long-acting release; mPFS = median progression-free survival; mTOR = mammalian target of rapamycin; NET = neuroendocrine tumor; NR = not reported; ORR = overall response rate; pNET = pancreatic neuroendocrine tumor; PRRT = peptide receptor radionuclide therapy; SSA = somatostatin analog; TKI = tyrosine kinase inhibitor.

Outcomes for patients with well-differentiated high-grade NETs demonstrate that those with a poor prognosis may benefit from more aggressive therapies early in their treatment journey, sparing more tolerable therapies like SSAs for those patients with indolent disease and a presumably longer survival [51]. Further prospective studies evaluating different treatment effects in patients with G3 NETs are needed to determine an optimal treatment strategy based upon the evaluation of potential adverse events and life expectancy, and, as the duration of NET treatment is often prolonged, patient quality of life (QoL) is always an important consideration [37,51]. The aim of this review is to summarize the latest literature on sequencing of therapies in high G2 (10% ≤ Ki-67 ≤ 20%) and G3 (Ki-67 > 20%) GEP-NETs and to identify the patient selection considerations for utilizing PRRT.

## 2. Treatment Sequencing Strategies for G2/G3 GEP-NETs

There is no established standard of care (SoC) for patients with higher G2 (Ki-67 ≥ 10% and ≤20%) and G3 (Ki-67 ≥ 20% and ≤55%) GEP-NETs and a paucity of high-quality evidence for treatment recommendations, especially in the 1L setting [31]. The optimal treatment for G3 NETs has yet to be described, therapy decisions need to be tailored to individual patients, and most of the current treatment regimens for G3 NETs are based on G1/G2 NET treatments and/or data from small retrospective studies [37,38].

### 2.1. Role of Imaging and Biomarkers in G2/G3 GEP-NET Treatment Planning

For many institutions, it is now standard practice to determine whether there is a more aggressive component of the disease that should be treated with an alternate therapy [52]; however, no validated biomarkers for GEP-NETs currently exist [53]. Utilization of molecular imaging, such as SSTR and fluorodeoxyglucose (FDG) positron emission tomography/computed tomography (PET/CT), and immunohistochemistry profiling in characterizing patients with advanced high-grade NENs can help to guide treatment selection and sequencing [54,55]. Chromogranin A can sometimes be used as a biomarker in patients with NETs; however, the American Joint Committee on Cancer (AJCC) and the National Comprehensive Cancer Network (NCCN) do not recommend its routine use as it can lack specificity and test values can fluctuate [1,56]. SSTR expression and hypermetabolic tumors assessed by [^68^Ga]Ga-DOTA-peptide PET and [^18^F]FDG PET scans, respectively, can be used for proper staging, prognosis evaluation, and treatment personalization, especially in patients with high-grade NETs [13,53,57,58,59]. SSTR imaging is currently considered the SoC for NENs [54], and, although not mandatory, obtaining dual [18F]FDG and [^68^Ga]Ga-DOTA-peptide PET scans that can help determine if hypermetabolic tumors also overexpress SSTRs, is well documented and widely accepted [52,60,61,62,63,64]; however, owing to geographical difference in resources and regulatory limitations, dual [^18^F]FDG and [^68^Ga]Ga-DOTA-peptide PET scans are yet to be universally applied [54].

**Figure 2:**
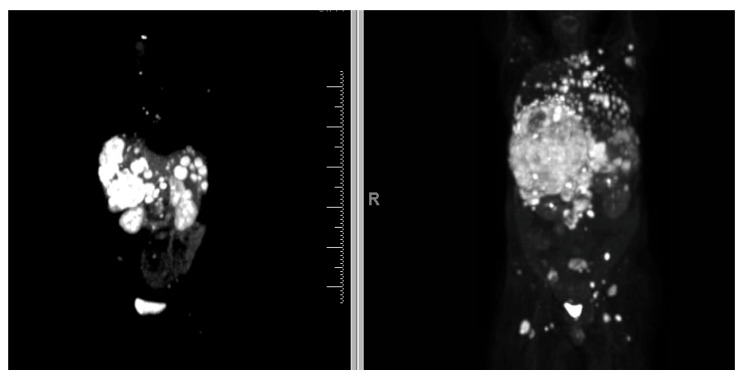
Comparative PET imaging in a patient with a G3 NET. (**Left**) [^68^Ga]Ga-DOTATATE PET with SSTR-positive hepatic metastases but SSTR-negative thoracic and retroperitoneal disease. (**Right**) [^18^F]FDG PET positive for metastatic hypermetabolic disease in retroperitoneum and thorax and relatively negative disease in liver. FDG = fluorodeoxyglucose; G = grade; NET = neuroendocrine tumor; PET = positron emission tomography; SSTR = somatostatin receptor.

The relationship between FDG and SSTR uptake is complex and can be heterogeneous; certain tumors co-express both markers, whereas others may express neither [65]. PET/CT images from two distinct scans in a patient with a G3 NET illustrate the heterogeneous nature of the disease. The [^68^Ga]Ga-DOTATATE PET/CT (Figure 2 Left) demonstrates SSTR-positive hepatic metastases but SSTR-negative thoracic and retroperitoneal disease, whereas the [^18^F]FDG PET/CT (Figure 2 Right) shows no significant FDG uptake in the liver but is positive for metastatic hypermetabolic disease in the retroperitoneum and thorax. This discordance between the two imaging modalities underlines the value of multimodal imaging for assessing high-grade NETs with heterogeneous presentation.

Additionally, metabolic response assessment using [^18^F]FDG PET/CT post-PRRT can provide a more accurate evaluation of treatment efficacy than anatomic imaging alone [65]. In a questionnaire-based survey that was distributed to the European Neuroendocrine Tumor Society Advisory Board Meeting attendees in 2022, the use of [^18^F]FDG PET/CT in G3 NETs as a baseline for response assessment, even in the case of fully matched lesions (detectable on both diagnostic CT and SSTR PET/CT), was favored by 74% [66]. In a retrospective study of patients with SSTR-positive G1/G2 NETs who received [^177^Lu]Lu-DOTATATE, baseline [^18^F]FDG PET positivity was found to be an independent predictive and prognostic factor and was associated with poor survival and shorter PFS compared with [^18^F]FDG-negative disease, especially in G2 NETs [67]. Furthermore, a complete metabolic response on [^18^F]FDG PET after PRRT in patients with baseline FDG-avid disease can be a strong prognostic indicator, even with residual anatomical or SSTR-avid lesions. In a cohort of patients treated with [^177^Lu]Lu-DOTATATE combined with 5-fluorouracil, the median PFS was not reached in patients with a complete metabolic response while those achieving a partial metabolic response had a median PFS of 17 months [65].

### 2.2. Treatment Sequence Practices

#### 2.2.1. Existing Guidelines

Most existing guidelines, which are based on older data and thus may not reflect latest advancements in the field, provide limited direction on treatment sequencing for higher-grade GEP-NETs [53,68,69,70]. European Society for Medical Oncology (ESMO) guidelines provide more detailed sequencing recommendations, depending on tumor origin, grade, and Ki-67 index [69]. However, a recent American Society of Clinical Oncology (ASCO) expert panel has noted that, particularly following disease progression, there is insufficient evidence available to inform recommendations for sequencing of therapy options [53]. Recommendations on treatment sequencing are mainly supported by low-level evidence as no randomized studies comparing active therapies are ongoing as of recently [52], thus treatment sequencing of PRRT varies by society and primary site [71]. Treatment recommendations of major societies for advanced, G2 and G3 GEP-NETs are summarized in Table 2. SSA monotherapy may be considered as 1L treatment in select cases of G3 GEP-NETs (i.e., SSTR-positive, low volume of disease, low tumor-related symptom burden, less rapid rate of growth) [53]. PRRT monotherapy or in combination with SSAs is recommended as a potential 2L treatment option for patients with SSTR-positive G3 GEP-NETs with characteristics such as less rapid rate of growth, and lower volume of disease [53]. PRRT is recommended by ESMO in G2 small intestine NETs (10% < Ki-67 < 15% or rapid growing) following everolimus and in G2 pNETs following chemotherapy [69]; however, a lower effectiveness of PRRT when performed after chemotherapy has been reported [72]. Although during the development of this review, none of the existing guidelines had reflected the recently published NETTER-2 data on 1L PRRT treatment of higher-grade GEP-NETs in their recommendations, a recent update in the NCCN guidelines has since included them, recommending 1L PRRT with [^177^Lu]Lu-DOTATATE in well-differentiated, SSTR-positive G2 GEP-NETs with Ki-67 ≥ 10% and clinically significant tumor burden [56]. According to North American Neuroendocrine Tumor Society (NANETs) expert consensus practice recommendations for high-grade NENs, liver-directed therapy is warranted in patients with G3 GEP-NETs with favorable disease (Ki-67 < 55%, no extrahepatic metastases) [17].

**Table 2:** Major societies’ higher-grade GEP-NET treatment recommendations.

Society	Midgut NETs		pNETs	
	**G2**	**G3**	**G2**	**G3**
American Society of Clinical Oncology (ASCO), 2023 [53]	1L: Octreotide/lanreotide 2L: PRRT (SSTR-positive) or everolimus (non-functional tumors)	1L: Octreotide/lanreotide (SSTR-positive, low volume) 2L: PRRT (SSTR-positive), everolimus (non-functional tumors), or chemotherapy (high Ki-67)	1L: Octreotide/lanreotide (SSTR-positive) or CAPTEM (higher-volume or SSTR-negative tumors) or everolimus or sunitinib 2L: PRRT (SSTR-positive), CAPTEM, everolimus, or sunitinib	1L: Octreotide/lanreotide (SSTR-positive, low volume) 2L: PRRT (SSTR-positive), everolimus (non-functional tumors), or chemotherapy (high Ki-67)
European Society for Medical Oncology (ESMO), 2020 [69]	1L: SSA or everolimus (Ki-67 > 10%) 2L: PRRT or everolimus (SSTR-negative) 3L: Everolimus (Ki-67 < 10%) FOLFOX, temozolomide (Ki-67 > 10%)	PRRT may be considered	1L: SSA (Ki-67 < 10%) or streptozotocin/5-FU/ CAPTEM/everolimus/ sunitinib (Ki-67 > 10%) 2L: Streptozotocin/5-FU/ CAPTEM/everolimus/sunitinib (Ki-67 < 10%) or PRRT (Ki-67 > 10%)	1L: CAPTEM, streptozotocin/5-FU 2L: Everolimus or sunitinib 3L: PRRT
North American Neuroendocrine Tumor Society (NANETS), 2017 [73], 2020 [70], 2023 [17]	1L: SSA (octreotide/lanreotide) 2L: [^177^Lu]Lu-DOTATATE or everolimus (SSTR-negative)	1L: Favorable disease: SSA (SSTR-positive), liver-directed therapy (if Ki-67 < 55%), PRRT (high SSTR expression), everolimus (if bowel origin) Aggressive disease: CAPTEM, FOLFOX/CAPEOX 2L: CAPTEM (if not used in 1L), FOLFOX, FOLFIRI	1L: SSA (octreotide/lanreotide) or chemotherapy, liver-directed therapy, everolimus, and/or sunitinib (SSTR-negative) 2L: Everolimus, sunitinib, or PRRT (SSTR-positive) Consider chemotherapy for progressive disease	1L: Favorable disease: SSA (SSTR-positive), liver-directed therapy, everolimus, sunitinib Aggressive disease: CAPTEM, FOLFOX/CAPEOX 2L: CAPTEM, FOLFOX/CAPEOX
National Comprehensive Cancer Network (NCCN), 2025 [56]	Preferred regimens: Cabozantinib, everolimus, [^177^Lu]Lu-DOTATATE (SSTR-positive and progression on SSAs), 1L [^177^Lu]Lu-DOTATATE (SSTR-positive, Ki-67 ≥ 10%), or SSAs (octreotide/lanreotide)	Preferred: Clinical trial Alternatives: Cabozantinib, chemotherapy, everolimus, SSAs (octreotide/lanreotide), pembrolizumab, [^177^Lu]Lu-DOTATATE (SSTR-positive)	Preferred regimens: Cabozantinib, everolimus, sunitinib, SSAs (octreotide/lanreotide), 1L [^177^Lu]Lu-DOTATATE (SSTR-positive, Ki-67 ≥ 10%), [^177^Lu]Lu-DOTATATE (SSTR-positive and progression on SSAs), or CAPTEM	Preferred: Clinical trial Alternatives: Cabozantinib, chemotherapy, everolimus, SSAs (octreotide/lanreotide), pembrolizumab, [^177^Lu]Lu-DOTATATE (SSTR-positive), sunitinib

1L = first line; 2L = second line; 3L = third line; 5-FU = fluorouracil; CAPEOX = capecitabine and oxaliplatin; CAPTEM = capecitabine and temozolomide; FOLFIRI = folinic acid, fluorouracil, and irinotecan; FOLFOX = folinic acid, fluorouracil, and oxaliplatin; G = grade; NET = neuroendocrine tumor; pNET = pancreatic neuroendocrine tumor; PRRT = peptide receptor radionuclide therapy; SSA = somatostatin analog; SSTR = somatostatin receptor.

#### 2.2.2. Factors That Influence Sequence of Treatment

a. Clinical Evidence

The level of clinical evidence on treatment modalities specifically for high-grade GEP-NETs is low. A 2023 systematic review examining the available data on the epidemiology, diagnosis, molecular changes, and treatment of G3 GEP-NETs found that most studies were retrospective and that the scientific evidence on those lacks significant quality [58]. An expert perspective on treatment sequencing for important clinical scenarios, ranging from local disease to high-volume metastatic NETs, was provided by Chauhan et al., in an effort to serve as a guide for clinicians making treatment sequencing decisions [74]. For patients with high-volume or symptomatic midgut NETs that progress after SSA, rapid introduction of PRRT was recommended [74]. Finally, when deciding the sequence of treatments, additional toxicities should be taken into consideration as well as their impact on the patient’s QoL [55].

b. Patient Characteristics and Preferences

Disease-related factors such as site of tumor origin, volume of disease, and patient-related characteristics including comorbidities, goal of treatment, and patient preferences should aid treatment sequencing strategies [55,74]. A shared decision-making approach that involves the patient while considering their values and preferences is recommended [53]. Before starting PRRT treatment, an expert panel agreed that specific and detailed oral and written information that includes notes about the purpose, procedure, and risk–benefit balance deriving from radiation use should be given to the patients [55]. Additionally, the unmet needs of patients with NENs should be identified with routinely evaluated patient-reported outcomes [55]. Naraev et al. noted that little information on general preferences of patients with advanced GEP-NETs is available to clinicians and this was identified as a barrier to how QoL data can be translated to aid clinical decision-making [75].

#### 2.2.3. Multidisciplinary Approach

The importance of a multidisciplinary approach of high-grade GEP-NET management is increasingly being highlighted. Optimization of treatment strategies can be supported by incorporating perspectives from all relevant medical specialties (e.g., medical oncology, interventional radiology, surgery, and nuclear medicine) [52]. It has been suggested that G3 NET treatment management should ideally be carried out in association with centers of excellence with involvement of a specialist NET multidisciplinary team [36]. When PRRT is implemented in the treatment strategy, dedicated clinical expertise is required due to the radioactive component of this type of therapy, and eligibility of patients with NETs for PRRT should also be discussed in an expert multidisciplinary team [76].

#### 2.2.4. Beyond Progression Treatment

Beyond 1L therapy, treatment options depend on the tumor primary site [52]. In pNETs, other systemic options beyond SSAs include everolimus, sunitinib, cabozantinib, temozolomide- or streptozocin-based chemotherapy regimens, or [^177^Lu]Lu-DOTATATE [27,52]. Options for other G3 NETs include folinic acid, fluorouracil, and irinotecan (FOLFIRI), folinic acid, fluorouracil, and oxaliplatin (FOLFOX), CAPTEM, cabozantinib, everolimus, sunitinib, and PRRT [54]. There is no compelling evidence that supports maintenance SSA after progression in patients with non-functioning tumors. However, in patients with hormonally functional tumors, octreotide or lanreotide is typically continued indefinitely for symptom management [52].

## 3. PRRT in Clinical Practice

Although the NETTER-1 trial enrolled patients with midgut NETs only, [^177^Lu]Lu-DOTATATE was approved for all GEP-NETs by the U.S. Food and Drug Administration and the European Medicines Agency on the basis of both the NETTER-1 trial and the Rotterdam database studies. Since NETTER-1 was conducted only in patients progressing on SSAs, the current recommendations are to use [^177^Lu]Lu-DOTATATE after progression and not in the 1L setting [52]. Based on consensus of a NANETs expert panel, it is reasonable to consider PRRT in patients with progressive G3 NET showing homogeneously high (avidity greater than liver) SSTR expression by imaging [17]. Although [^177^Lu]Lu-DOTATATE was traditionally administered in specialized institutions, commercialization of PRRT has now enabled smaller hospitals and day clinics to also administer these therapies [6]. Additionally, since its approval, both prospective and retrospective studies have shown that PRRT treatment with [^177^Lu]Lu-DOTATATE can lead to an improved or at least stable health-related (HR)QoL in several domains [77,78,79]. In a NETTER-1 sub-analysis of the HRQoL of patients treated with [^177^Lu]Lu-DOTATATE + octreotide LAR vs. those treated with high-dose octreotide, clinically significant differences in median time to deterioration were observed in domains such as global health status (28.8 months vs. 6.1 months) and physical functioning (25.2 months vs. 11.5 months) [77].

### 3.1. Patient Selection Criteria

For a patient with GEP-NETs, SSTR uptake in all lesions is considered a requirement for PRRT eligibility [76], although of note is that SSTR expression varies by primary site [52]. A systematic checklist for assessing a patient’s PRRT eligibility has been proposed by Burkett et al. The checklist includes clinical and treatment history, tumor grade, and stage, imaging findings that demonstrate sufficient tumor SSTR uptake, and laboratory values that for sufficient bone marrow reserves, kidney function, and liver function [80]. Evaluation of the pretreatment laboratory values such as creatinine clearance, hemoglobin, leukocytes, platelet count, bilirubin, alanine aminotransferase, and aspartate aminotransferase is required, since PRRT can induce toxicity [76]. Decreased renal function and extensive hepatic and/or bone disease may limit PRRT indication [81]. It should be noted, however, that recent studies suggest that, even in mild-to-moderate renally compromised patients, PRRT appears to be well tolerated and lacks long-term nephrotoxicity [82,83]. Patients with NETs and existing mesenteric or peritoneal disease appear to be at high risk for developing bowel obstruction when treated with PRRT, as evidenced by the temporal relationship between treatment with [^177^Lu]Lu-DOTATATE and bowel obstruction that was observed in a small retrospective study. Treating physicians and patients should be aware of this potential complication in high-risk patients [84]. Combinations with radiation sensitizers, DNA-repair inhibitors, or immune-activating agents can be utilized in patients with low SSTR expression or those who respond poorly to [^177^Lu]Lu-DOTATATE, to improve its efficacy [6].

### 3.2. PRRT as 1L in G2/G3 GEP-NETs (10% ≤ Ki-67 ≤ 55%)

#### 3.2.1. Rationale

PRRT has been reserved for later lines of therapy, predominantly used in advanced NENs when all other therapies fail [85], with the exception of select patients with a high tumor burden that require early aggressive treatment [52]. Multiple factors such as tumor grade and origin, SSTR expression, disease burden, and clinical symptomatology influence the choice of optimal 1L treatment as well as the position of PRRT in the treatment sequence [16,86]. Until NETTER-2, no other phase 3 trials have been completed comparing 1L PRRT with other standard, approved systemic, or liver-directed therapies.

#### 3.2.2. Safety

In NETTER-2, commonly reported adverse events included nausea, diarrhea, and hematologic toxicities (i.e., thrombocytopenia); however, no unexpected safety signals were observed compared with other trials evaluating PRRT in GEP-NETs [31]. Acute side effects such as nausea and vomiting can be primarily attributed to the co-infusion of positively charged amino acids administered to provide renal protection [87]; however, compounded arginine/lysine formulations have largely eliminated this issue [88,89,90]. G ≥ 3 thrombocytopenia, anemia, neutropenia, and leukopenia were reported in < 2% of patients [31]. Similarly, in the NETTER-1 trial that evaluated 2L PRRT in midgut NETs, G3/G4 neutropenia, thrombocytopenia, and lymphopenia observed in < 10% of [^177^Lu]Lu-DOTATATE–treated patients [46]. In the OCLURANDOM study of patients with progressive, advanced, pancreatic NET, G3/G4 events were experienced by fewer patients treated with [^177^Lu]Lu-DOTATATE (56%) than with sunitinib (72%). Additionally, [^177^Lu]Lu-DOTATATE was better tolerated, with 56% compared with 13% of sunitinib-treated patients reporting “not at all” side effects from treatment at 36 weeks [49]. In a retrospective cohort study of 149 patients with G3 GEP-NENs who received PRRT, similar rates of G3/G4 toxicities among different lines of treatment were observed [91]. In the COMPETE study, which evaluated the efficacy and safety of [^177^Lu]Lu-edotreotide vs. everolimus in patients with inoperable, progressive, SSTR-positive G1/G2 GEP-NETs (Ki-67 ≤ 20%), a lower proportion of patients treated with [^177^Lu]Lu-edotreotide presented with adverse events than those treated with everolimus [48]. Severe long-term toxicities such as acute leukemia or myelodysplastic syndrome (MDS) have been reported in <~1–4% of patients treated with [^177^Lu]Lu-DOTATATE [31,46,92,93,94]. Of note, however, are the observed higher rates (8–20%) of MDS/acute myeloid leukemia in patients with GEP-NETs treated with PRRT who also received chemotherapy, either in combination or sequentially, than those reported for PRRT alone [89,95]. Chemotherapy has been demonstrated to be associated with excess MDS/acute myeloid leukemia risk for several types of solid tumors [96] and this is a risk worth considering when positioning PRRT in treatment sequencing decisions.

#### 3.2.3. Efficacy

In NETTER-2 a significant PFS benefit of [^177^Lu]Lu-DOTATATE plus octreotide 30 mg LAR vs. high-dose octreotide 60 mg LAR was observed (median PFS 22.8 months vs. 8.5 months) [31]. Observed response rate in the [^177^Lu]Lu-DOTATATE group was 43.0% vs. 9.3% in the high-dose octreotide group [31]. In a NETTER-2 subgroup analysis that examined [^177^Lu]Lu-DOTATATE efficacy by NET grade and NET origin, high observed response rates were seen in patients with G3 NETs (48.1%) and pNETs (51.2%) [97]. Another NETTER-2 sub-analysis that assessed time to response, found that the median time to response among 65 responders in the [^177^Lu]Lu-DOTATATE arm was 5.7 months (interquartile range 4.1–8.3) [98]. In the OCLURANDOM study comparing [^177^Lu]Lu-DOTATATE with sunitinib in patients with progressive advanced pancreatic NET, median PFS was longer with [^177^Lu]Lu-DOTATATE (20.7 months) than with the comparator treatment (11.0 months) [49]. In a retrospective, multicenter cohort study of 149 patients with G3 GEP-NENs who received PRRT, similar response rates were observed (42%) while the median PFS was 14.0 months. However, interestingly, no differences in these variables were evident among lines of treatment (1L vs. 2L vs. later-line) [91]. Recently published results of the COMPETE trial demonstrated a significant PFS benefit of [^177^Lu]Lu-edotreotide vs. everolimus (median PFS 23.9 months vs. 14.1 months) in patients with G1/G2 GEP-NETs [48].

#### 3.2.4. PRRT Retreatment Practices

Off-label use of PRRT retreatment in progressive NETs is common global practice [99], though it has been recommended that it should be limited to specific clinical studies [55]. However, evidence of the antitumoral effects of PRRT retreatment exists with several retrospective cohort studies having reported both safety and efficacy [76,99]. Reported median PFS in patients who underwent PRRT retreatment ranged from 11.0 to 14.0 months [100,101,102]. Safety profiles of PRRT retreatment similar to the initial PRRT treatment have also been reported [100,102]. The NET RETREAT trial is currently exploring PRRT retreatment efficacy and safety in a randomized fashion ([^177^Lu]Lu-DOTATATE retreatment vs. everolimus; NCT05773274) [103]. The ACTION-1 trial is comparing treatment with the ^225^Ac-labeled SSA RYZ101 ([^225^Ac]Ac-DOTATATE) vs. SoC (everolimus, sunitinib, octreotide, or lanreotide) in patients with G1/G2 GEP-NETs who have progressed after treatment with a ^177^Lu-labeled SSA (NCT05477576) [104].

## 4. Future Directions

### 4.1. Investigation of New Isotopes in PRRT

The preferred β^−^-emitting agent for NET PRRT is [^177^Lu]Lu-DOTATATE, largely due to its reduced nephrotoxicity potential compared with [^90^Y]Y-DOTATOC [80]. The differences in tissue permeation of the β radiation have been suggested to be central in causing kidney damage, as β^−^ radiation emitted by ^90^Y penetrates tissue to a depth of 11 mm maximum while Lu-177 to a depth of 2 mm maximum [80]. However, it has been postulated that the increased tissue penetration of Y-90 β^−^ emission compared with Lu-177 may be advantageous for larger tumors [80]. Better OS has also been observed with a combination of Y-90 and Lu-177 PRRT, compared with either radionuclide alone [100]. A prospective pilot study comparing combination [^177^Lu]Lu-/[^90^Y]Y-DOTATOC treatment with [^177^Lu]Lu-DOTATOC monotherapy in FDG-positive NENs, found that the combination therapy more effectively reduced FDG uptake, particularly in pancreatic and unknown primary tumors, with responses independent of Ki-67 [105].

Other radionuclides besides Y-90 and Lu-177, such as the α emitters Ac-225, Tb-161 and Pb-212 are also being studied [80,106]. By emitting much larger particles (two protons and neutrons) with higher linear energy transfer over an ultrashort particle range, α emitters can induce double-strand DNA damage and a higher level of cytotoxicity with an improved therapeutic index [52]. Several clinical trials investigating α-emitting PRRT such as [^212^Pb]Pb-DOTAMTATE, [^212^Pb]Pb-VMT-α-NET, and [^225^Ac]Ac-DOTATATE as a possible alternative to β^−^-emitting PRRT are ongoing (NCT03466216, NCT05636618, and NCT06732505) [107,108,109].

### 4.2. Other Ongoing PRRT Studies

Data from other PRRT trials will shed light on the earlier positioning of PRRT in the treatment algorithm [106]. Trials including COMPOSE ([^177^Lu]Lu-edotreotide vs. chemotherapy; NCT04919226) and PRRT combination trials ([^177^Lu]Lu-DOTATATE + nivolumab; NCT04525638, [^177^Lu]Lu-DOTATATE + triapine; NCT05724108, [^177^Lu]Lu-DOTATATE + peposertib) are ongoing.

#### 4.2.1. COMPOSE ([^177^Lu]Lu-Edotreotide vs. CAPTEM, Everolimus, FOLFOX)

The COMPOSE study is currently evaluating the early use (including 1L) of PRRT with [^177^Lu]Lu-edotreotide vs. best SoC (either chemotherapy [CAPTEM or FOLFOX] or everolimus) in patients with well-differentiated high G2 and G3 (Ki-67 index 15–55%) GEP-NETs (NCT04919226).

#### 4.2.2. NCT04525638 ([^177^Lu]Lu-DOTATATE + Nivolumab)

Clinical evidence on potential synergistic effects associated with combining treatments is scarce [6]. A possible synergistic effect of PRRT radiation, which is thought to increase tumor antigen release and promote immune cell infiltration, could enhance the overall therapeutic efficacy of an immunotherapy combination [6]. A phase 2 single-arm trial evaluating the preliminary efficacy of [^177^Lu]Lu-DOTATATE in combination with nivolumab in G3 GEP-NETs is ongoing (NCT04525638).

#### 4.2.3. NCT05724108 ([^177^Lu]Lu-DOTATATE + Triapine)

Triapine is a ribonucleotide reductase inhibitor and promising preclinical data support its role as a radiosensitizer in pNET models [110]. A first-in-human phase 1 clinical trial of [^177^Lu]-DOTATATE + triapine in SSTR-positive GEP-NETs was conducted and has led to a currently ongoing phase 2 study that is comparing this combination vs. [^177^Lu]Lu-DOTATATE alone (NCT05724108) [111].

#### 4.2.4. [^177^Lu]Lu-DOTATATE + Peposertib

Peposertib is a selective inhibitor of DNA-dependent protein kinase that is involved in DNA repair mechanisms and has been used as a radiosensitizer in preclinical NET models. A phase 1 trial evaluating [^177^Lu]Lu-DOTATATE in combination with peposertib in patients with SSTR-positive GEP-NETs is ongoing [112].

#### 4.2.5. PRRT + Chemotherapy Combination

Some evidence indicates that concurrent or sequential administration of cytotoxic chemotherapy may increase the risk of MDS or acute leukemia [52]. However, smaller studies that evaluated PRRT in combination with chemotherapy have demonstrated promising safety and survival outcomes [113,114].

## 5. Conclusions

### 5.1. How Can the Data from Recent 1L Studies of PRRT Impact the Treatment Sequencing Guidelines?

Until the recent NETTER-2 trial, data on PRRT for G3 NETs were scant and primarily retrospective, and while patients with G2 NETs were included in some of the previously published randomized controlled trials, there are relatively few data to be found with regard to the 10–20% Ki-67 subgroup [16]. NETTER-2 results started filling the evidence gap for PRRT treatment in high-grade GEP-NETs highlighted in treatment guidelines and might aid treatment decision-making for these patients [31]. Given the subgroup analyses in NETTER-2, PRRT can now be considered as a potential 1L treatment for SSTR-positive G3 GEP-NET patients [16,97]. However, even though the NETTER-2 PFS results demonstrate PRRT superiority, it must be noted that OS data from this trial are not yet mature. Therefore, it is important to exercise caution in interpreting these results.

### 5.2. Clinical Practice Recommendations/Expert Opinions from the Authors

**Figure 3:**
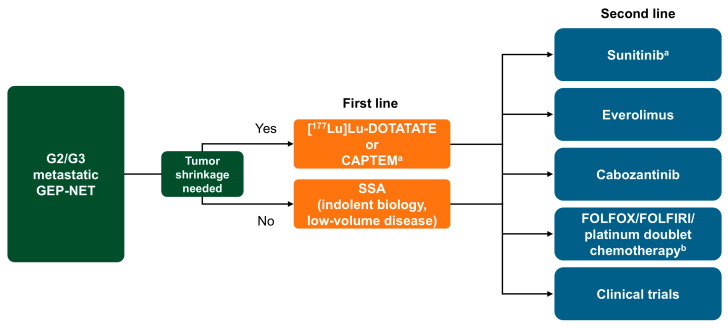
Suggested treatment algorithm for the management of G2/G3 metastatic GEP-NETs. ^a^ In pNETs; ^b^ In G3 GEP-NETs. CAPTEM = capecitabine and temozolomide; FOLFIRI = folinic acid, fluorouracil, and irinotecan; FOLFOX = folinic acid, fluorouracil, and oxaliplatin; G = grade; GEP-NET = gastroenteropancreatic tumor; pNET = pancreatic neuroendocrine tumor; SSA = somatostatin analog.

After NETTER-2, there is robust randomized data that support PRRT as 1L therapy in NETs with more aggressive biology. The authors recommend that all patients with well-differentiated, higher G2 and G3 NETs are evaluated for baseline SSTR expression using [^68^Ga]Ga-DOTA-peptide PET; addition of [^18^F]FDG PET/CT to exclude any mismatch lesions could also be considered. If positive, PRRT should be considered a 1L treatment option, especially in patients who are clinically stable and have access to PRRT. Pregnancy is a contraindication, and caution is advised in patents with glomerular filtration rate 45–55, very high liver bulk but normal liver function tests, or extensive prior bone marrow radiation exposure (> 50%) from external beam radiation therapy. Patients with low-volume, indolent disease may also benefit from SSA; however, close monitoring is recommended, as long-term SSA use has been associated with increased risk of late-onset complications related to cholelithiasis, including acute cholecystitis, gangrenous cholecystitis, or intestinal occlusion [115], also considering that many higher G2 and G3 NETs show aggressive growth patterns. For patients in visceral crisis, in need of urgent treatment, or with no access to [^68^Ga]Ga-DOTA-peptide PET imaging, chemotherapy might be preferred, especially in those with pNETs and G3 epNETs. CAPTEM has been shown to be highly beneficial in pNETs. Good choices for 2L or therapy are CAPTEM and cabozantinib. A suggested treatment algorithm is shown in Figure 3. This manuscript is largely centered around systemic management of metastatic NETs; however, there may be a role of surgical debulking and liver-directed therapy in select, well-differentiated NET patients. A robust discussion at a multidisciplinary NET tumor board can be beneficial in complex cases.

### 5.3. Further Research Needed for G2/G3 GEP-NET Management

Studies should focus more on the identification of biomarkers that can predict response to PRRTs in patients with G2 and G3 GEP-NETs. This will not only aid personalized treatment planning but also allow for better monitoring of these patients thus improving their prognosis. There is also a need for more long-term follow-up studies after treatment, to assess the durability of response, PFS, and OS in patients with GEP-NETs. These long-term insights can be instrumental in refining treatment protocol and improving patient care. Research should also be directed toward developing new therapeutic agents specific to higher-grade tumors (G2/G3), that can either be used in combination with existing therapies or serve as standalone treatments for GEP-NETs. These novel agents could offer better therapeutic results and improved QoL in patients with high-grade NETs.
